# Supplementary material for: Inferring the regional distribution of Visceral Leishmaniasis incidence from data at different spatial scales
Source: Commun Med (Lond). 2024 Nov 20;4:240. doi: 10.1038/s43856-024-00659-9 (PMC11579291; doi:10.1038/s43856-024-00659-9)
Supplement: Supplementary file 2 — Supplementary Information [file 43856_2024_659_MOESM2_ESM.pdf]

Supplementary Information

Supplementary Methods: Priors

Fixed effects

$$\beta_0 \sim N(-4, 8)$$

$$\beta_k \sim N(0, 0.4)$$

Random effects - block-level

$$u_i \sim N(0, \sigma_u^2)$$

where σ_u is assigned a penalised complexity prior [1] such that

$$P[\sigma_u > 0.1] = 0.01$$

This shrinks the model towards the simplest case of no block-specific variation, except that which arises from the fixed covariate effects.

Random effects - Spatial field

The spatial random field is parameterised by the scale σ and range ρ of the Matern covariance function, which are again assigned penalised complexity priors as in [2],

$$P[\sigma > 2] = 0.01$$

$$P[\rho < 0.1] = 0.01$$

This shrinks the field towards the simplest 'flat' case, with zero variation and infinite range.

Supplementary Figures

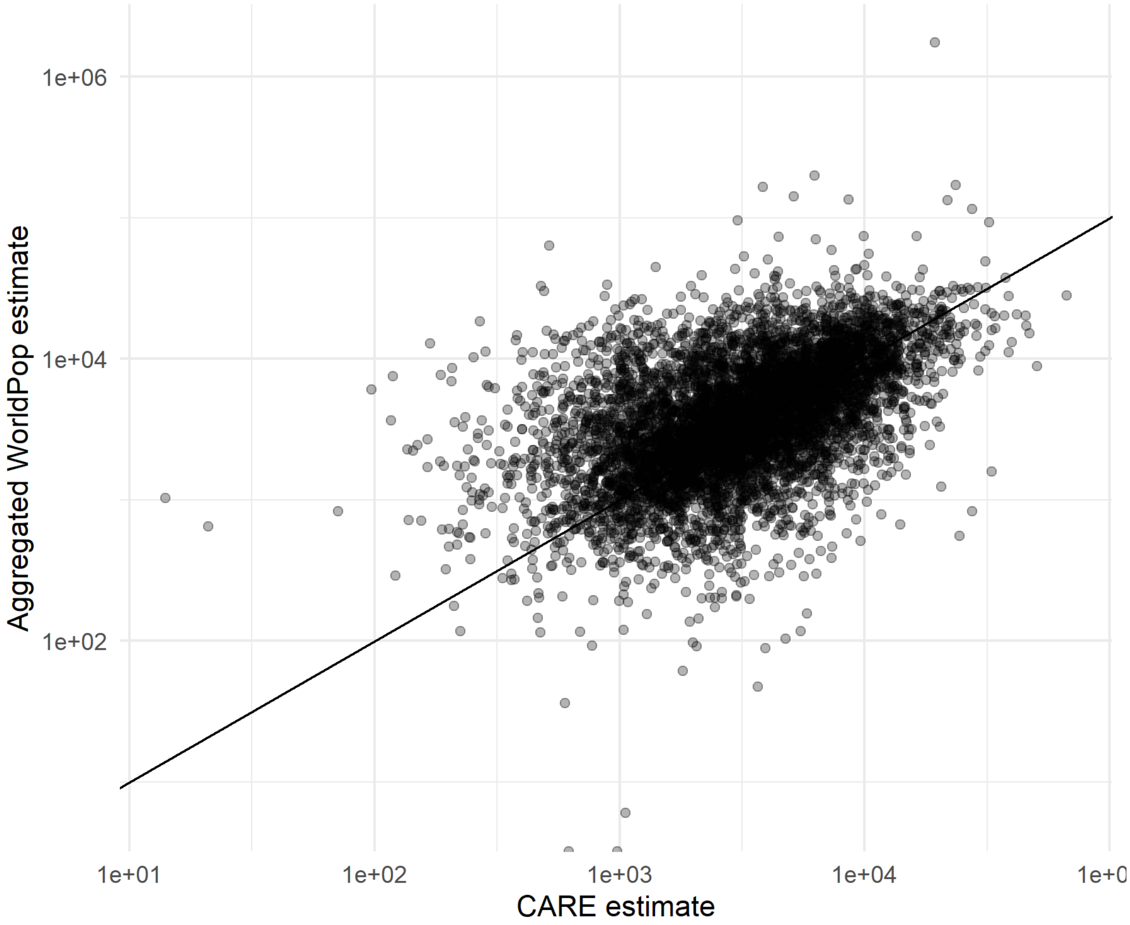

Figure S1: Village population estimates obtained by CARE field teams through routine surveillance versus estimates for the same villages aggregated from WorldPop raster data.

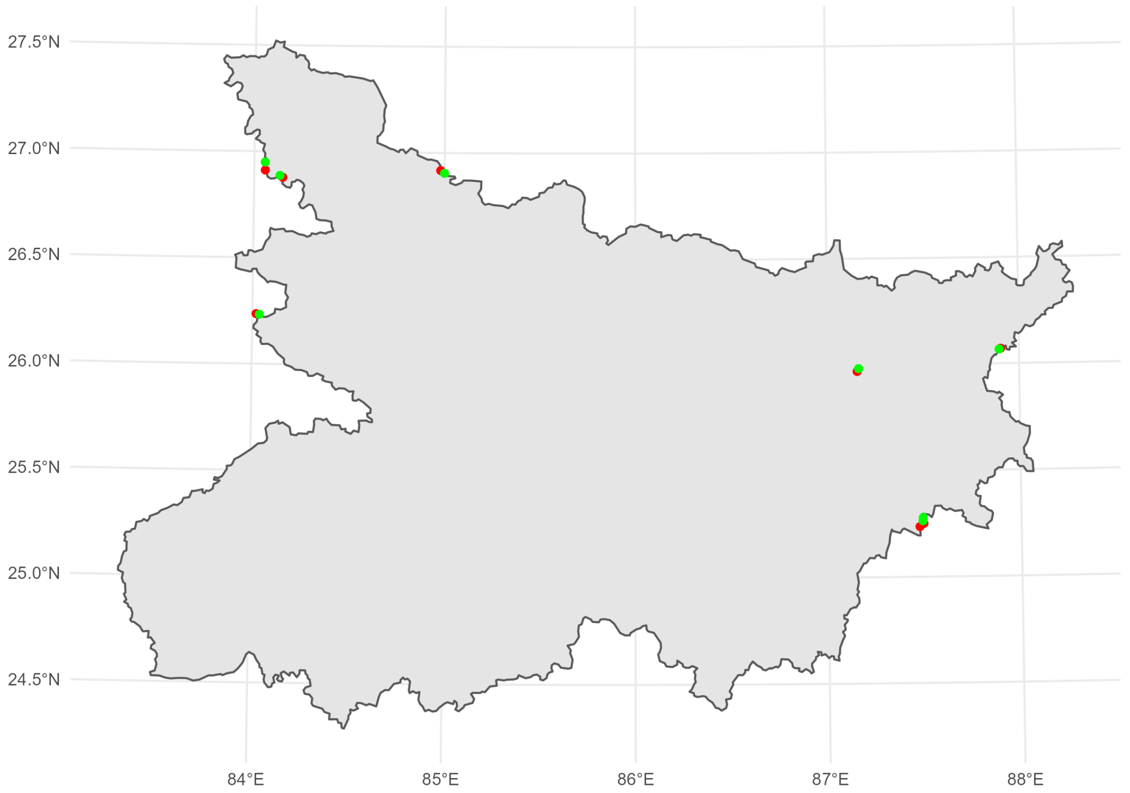

Figure S2: Village GPS locations which did not initially fall within a village polygon. Original locations are shown in red with snapped locations in green.

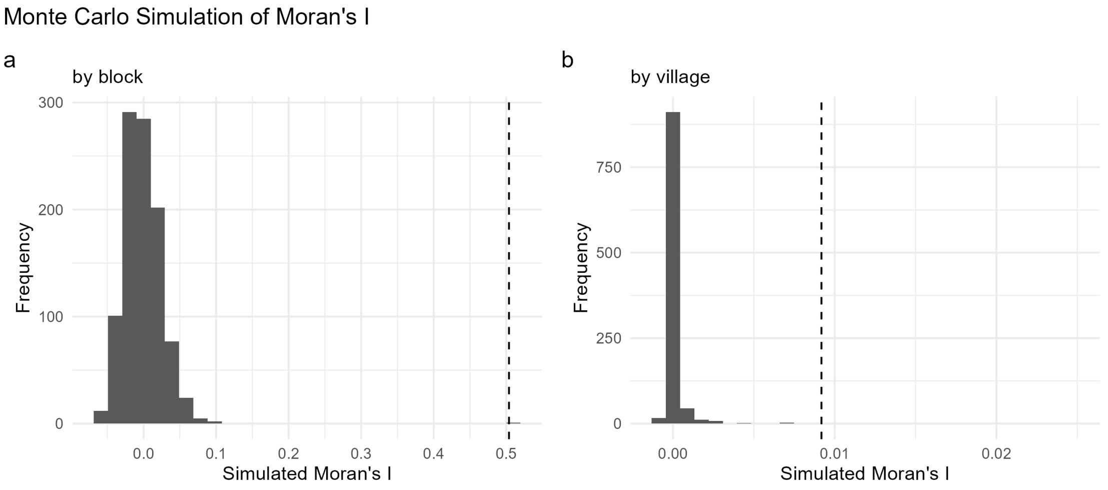

Figure S3: Evaluation of Moran's I statistic for state-wide VL incidence at the block (left) and village (right) level. The dashed vertical line illustrates the observed value, alongside the distribution of 999 values simulated under the assumption of complete spatial randomness.

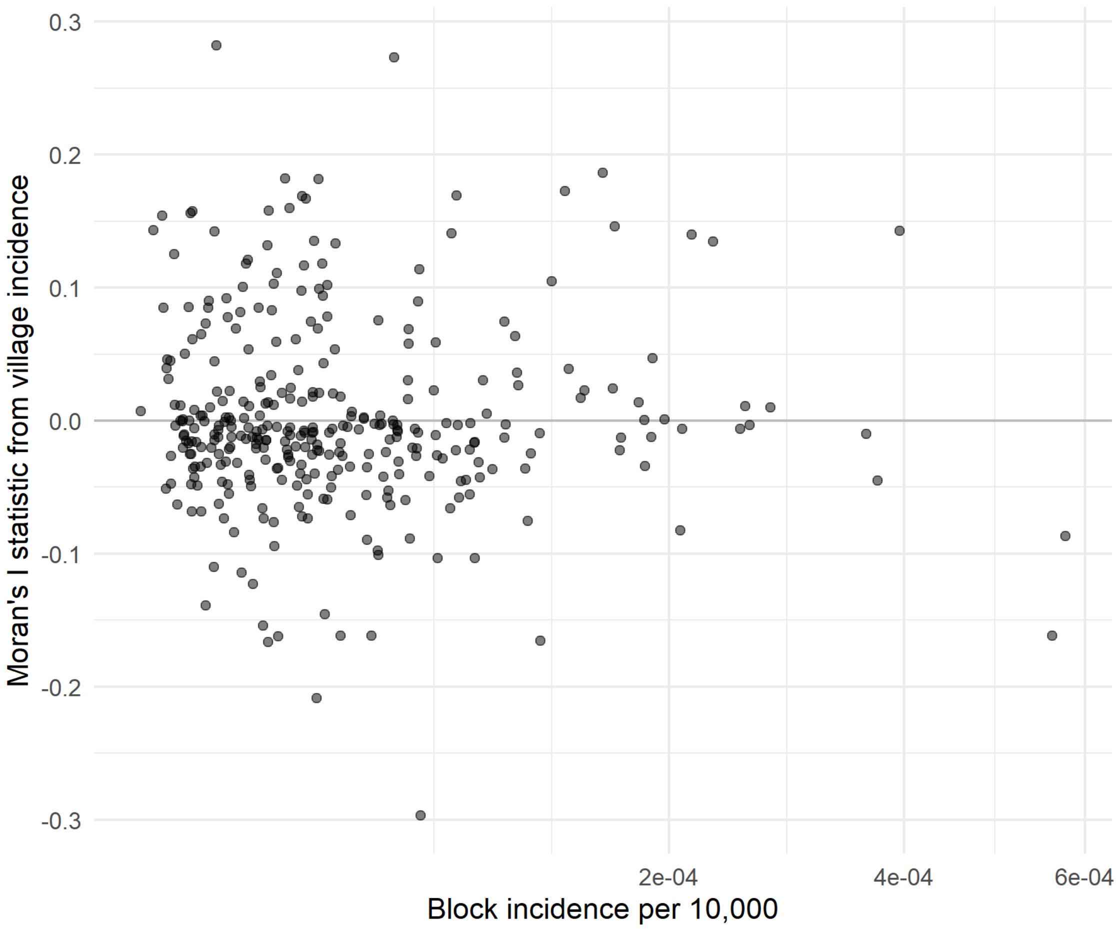

Figure S4: Moran's I statistic calculated across constituent villages of each non-zero incidence block, and plotted against the block's overall incidence rate. There does not appear to be any trend between the magnitude of the statistic to the level of block endemicity.

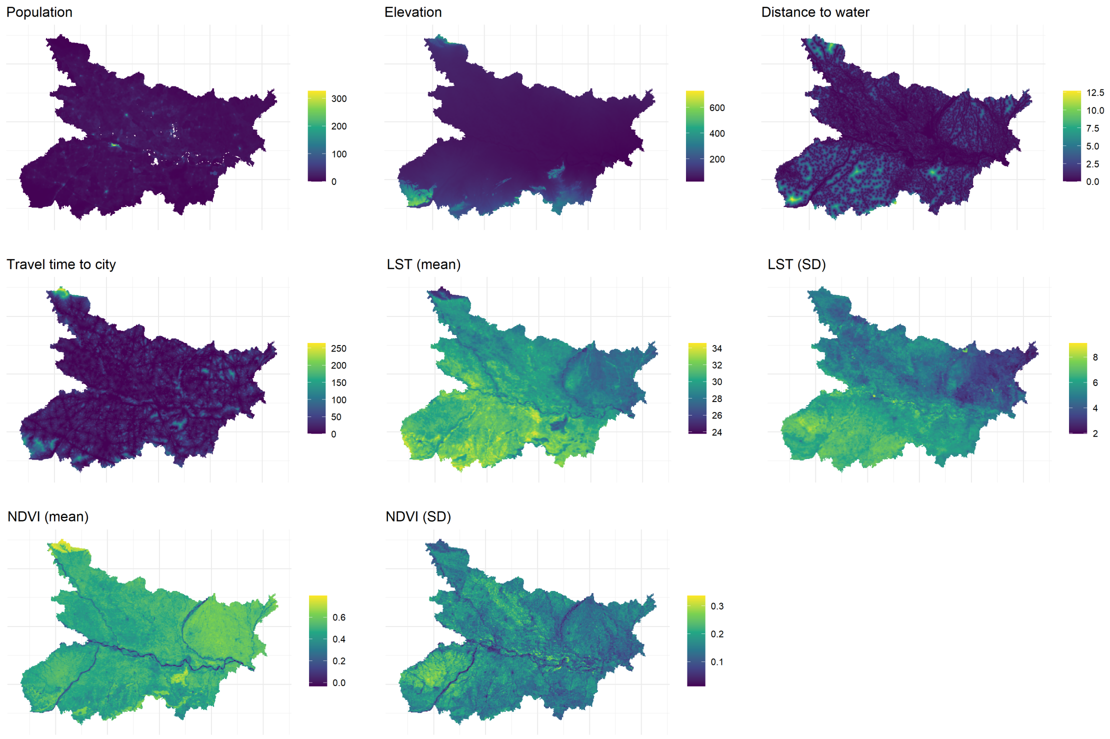

Figure S5: Visualisations of population and covariate raster data included in the disaggregation model (resolution 1km).

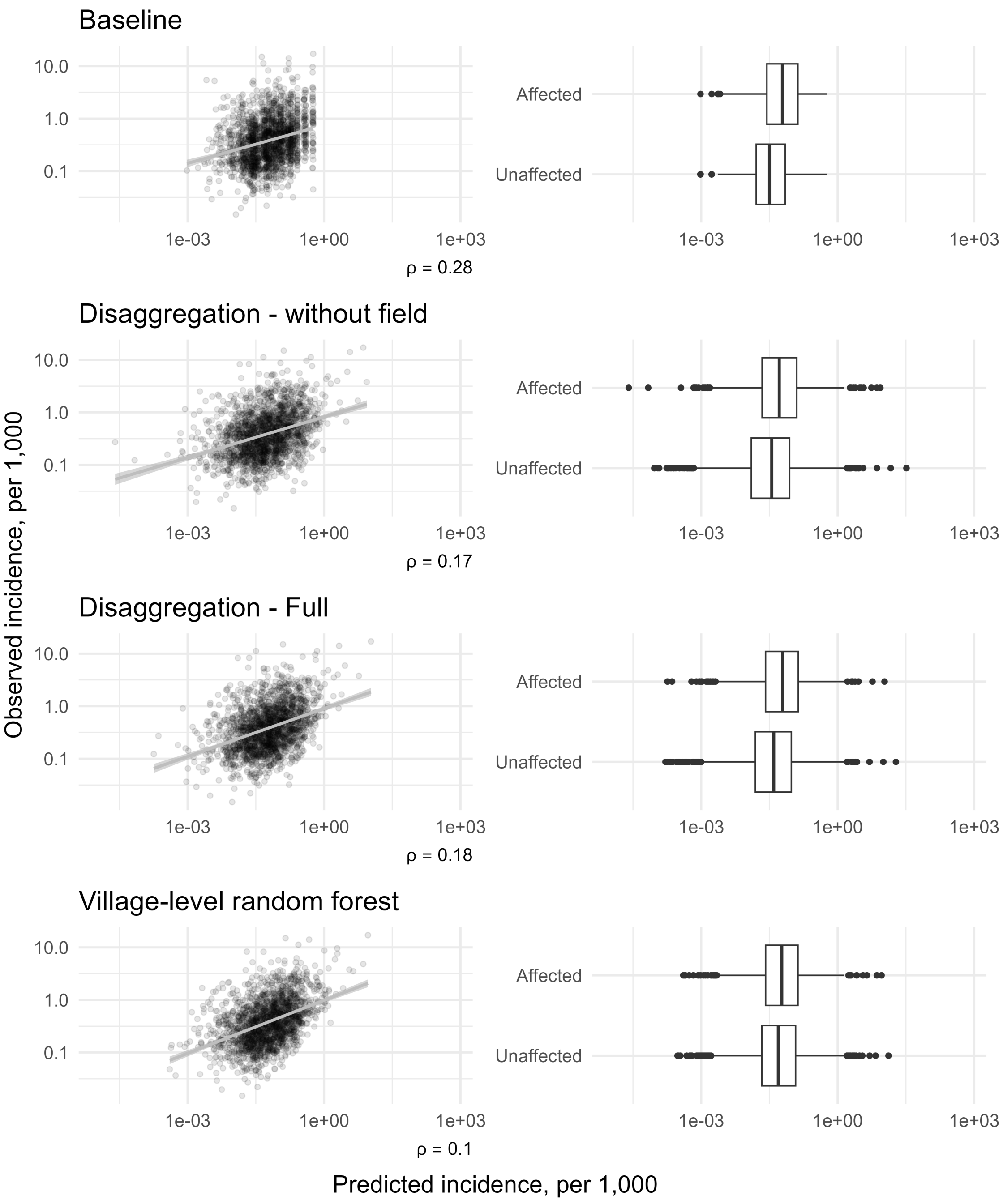

Figure S6: Comparison of predicted to observed village incidence rates, relative to locally-informed population estimates from CARE India as opposed to aggregation of WorldPop estimates to village polygons. Scatter plots only include affected villages, with non-zero observed and predicted incidence. Grey lines illustrate a simple linear trend (and 95% CI) of observed against predicted. Box plots include only villages with non-zero predicted incidence, to allow log transformation of the x-axis (note the different x-axis scales between models).

Supplementary References

[1] Daniel Simpson et al. "Penalising Model Component Complexity: A Principled, Practical Approach to Constructing Priors". EN. In: *Statistical Science* 32.1 (Feb. 2017). Publisher: Institute of Mathematical Statistics, pp. 1–28. ISSN: 0883-4237, 2168-8745. DOI: 10.1214/16-STS576. URL: https://projecteuclid.org/euclid.ss/1491465621 (visited on 02/01/2021).

[2] Geir-Arne Fuglstad et al. "Constructing Priors that Penalize the Complexity of Gaussian Random Fields". In: *Journal of the American Statistical Association* 114.525 (Jan. 2019), pp. 445–452. ISSN: 0162-1459. DOI: 10.1080/01621459.2017.1415907. URL: https://doi.org/10.1080/01621459.2017.1415907 (visited on 06/19/2024).
